# Learning Curve of Robot-Assisted Thymectomy: Single Surgeon's 7-Year Experience

**DOI:** 10.3389/fsurg.2022.860899

**Published:** 2022-08-08

**Authors:** Elisa Meacci, Dania Nachira, Maria Teresa Congedo, Leonardo Petracca-Ciavarella, Maria Letizia Vita, Venanzio Porziella, Marco Chiappetta, Filippo Lococo, Diomira Tabacco, Elizabeth Katherine Anna Triumbari, Stefano Margaritora

**Affiliations:** ^1^ Dipartimento di Medicina e Chirurgia Traslazionale, Università Cattolica del Sacro Cuore, Roma, Italy; ^2^ Nuclear Medicine Unit, TracerGLab, Department of Radiology, Radiotherapy and Hematology, Fondazione Policlinico Universitario A. Gemelli, IRCCS, Roma, Italy

**Keywords:** robot-assisted thymectomy, thymectomy, rat, myasthenia gravis, learning curve, CUSUM analysis

## Abstract

**Background:**

Robot-assisted thymectomy (RAT) has rapidly emerged as the preferred approach over open trans-sternal or video-assisted thoracoscopy for the surgical treatment of thymomas and non-thymomatous myasthenia gravis (MG). The aim of this study was to describe and discuss the learning curve (LC) of a single surgeon performing 113 consecutive RATs.

**Methods:**

A single-center retrospective analysis of prospectively collected clinical data was performed on all patients who had been operated on by the same surgeon in an RAT setting between October 2013 and February 2020. The cumulative sum (CUSUM) analysis of the operative time was used to define the completion of the learning curve (CLC) in RAT. The CLC was separately calculated for myasthenic patients, non-myasthenic patients, and docking time.

**Results:**

In myasthenic patients, the CLC cut-off was found in 19 patients. Considering the CLC cut-off of 19 patients, the mean operative time in phase 1 (first 19 cases) was 229.79 ± 93.40 min, while it was 167.35 ± 41.63 min in phase 2 (last 51 cases), p≪0.001. In non-myasthenic patients, the CLC cut-off was found in 16 cases. The mean operative time in phase 1 (first 16 cases) was 277.44 ± 90.50 min, while it was 169.63 ± 61.10 min in phase 2 (last 27 cases), *p* = 0.016. The LC for docking time was reached at 46 cases, recording a significant reduction of time after the first phase (28.09 ± 5.37 min vs. 19.75 ± 5.51 min, p≪0.001). The intraoperative and 30-day mortality were null in all phases of the LC in both myasthenic and non-myasthenic patients. There were no differences between the two phases of the LC in terms of blood loss, duration of postoperative drainage, and postoperative stay in both myasthenic and non-myasthenic groups. However, significantly higher hospital readmission at 30 days post surgery was recorded for myasthenic patients operated on during the first phase of the LC (2 cases vs. 0, *p* = 0.02).

**Conclusions:**

According to our data, LC in RAT seems to be steep, and RAT confirms to be safe even before reaching CLC.

## Introduction

Radical thymectomy is accepted as the surgical standard for the treatment of thymomas and represents a consistent therapeutic option to increase the probability of remission/improvement of neurological symptoms in non-thymomatous myasthenia gravis (MG) (1). Median sternotomy has historically represented the standard approach to thymectomy and is still considered the “gold standard” technique in the treatment of thymic disease.

However, the benefits of minimally invasive surgery in comparison with open sternotomy have been well established; many studies have demonstrated shorter postoperative stay, less pain, decreased blood loss, and equivalent oncological and functional outcomes after minimally invasive thymectomy (2–6).

The recently introduced robotic platform, which offers an excellent 3D visualization of the operating field, improved dexterity related to wristed instrument tips, and tremor filtration, makes the thymic dissection easier in a narrow space such as the mediastinum, driving a higher number of surgeons to consider thymectomy as the most appealing and attractive thoracic procedure to be performed by robotics.

The disadvantages of the robotic system include the initial costs and maintenance requirements, and, from a technical point of view, the lack of tactile feedback and its immobility once fixed into position. These limitations can lengthen the operating time in case of poor positioning, and, even worse, make the management of major vascular injuries challenging. In light of this and taking into account that the LC can have a substantial impact on surgical metrics, clinical outcomes and cost–benefit decisions, the need for standardized surgical education and proper robotic surgery training has been advocated (7).

Despite the growing interest in RAT, there is a paucity of clinical data describing the associated LC. To date, only three small observational studies have reported an LC analysis in RAT (8–10). Due to the small number of studies in the literature and some heterogeneities in the case series described, the learning curve for RAT has not been standardized yet.

At Fondazione Policlinico Universitario A. Gemelli IRCCS (Rome, Italy), RAT has been routinely performed to treat thymic disease since 2013. This study aims to evaluate the RAT LC of a single surgeon with the cumulative summation (CUSUM) analysis (11) in this setting.

## Materials and Methods

A single-center retrospective revision and analysis of prospectively collected clinical data were performed on all patients who had undergone RAT between October 2013 and February 2020. All RATs were performed by the same thoracic surgeon (E.M.) without previous experience in robotic procedures, except for four lung resections and one thymectomy performed under tutoring during the training phase.

### Patient Selection

Patients considered eligible for this study were adults with thymomatous or non-thymomatous MG and thymomatous patients without MG undergoing elective RAT. The following criteria were met for the selection of thymomatous patients suitable for RAT: no signs of the infiltration of surrounding structures, except resectable lung parenchyma/pericardium or mediastinal fat tissue. Patients with great vessel infiltration were excluded from the study cohort.

### Preoperative Assessment

Preoperative assessments included blood tests, electrocardiogram (or other cardiological examinations, when required), chest computed tomography, respiratory function tests, and neurological evaluation. All patients provided informed consent for the surgical intervention and processing of their clinical data at hospital admission. The research was conducted according to the recommendations outlined in the Declaration of Helsinki.

This study was evaluated by the Institutional Review Board (IRB) of the “Università Cattolica del Sacro Cuore,” and received IRB approval: ID 4112.

### Surgical Technique and Perioperative Management

All thymectomies were performed under general anesthesia with double-lumen ventilation tubes.

Our standard approach was the three-trocar left-sided robotic thymectomy technique (except in those cases where the thymic lesion was almost totally located on the right edge of the mediastinum) using the da Vinci Xi (Intuitive Surgical, Sunnyvale, CA, USA) robotic system, except for the first five procedures, where we used the SI system.

**Figure 1:**
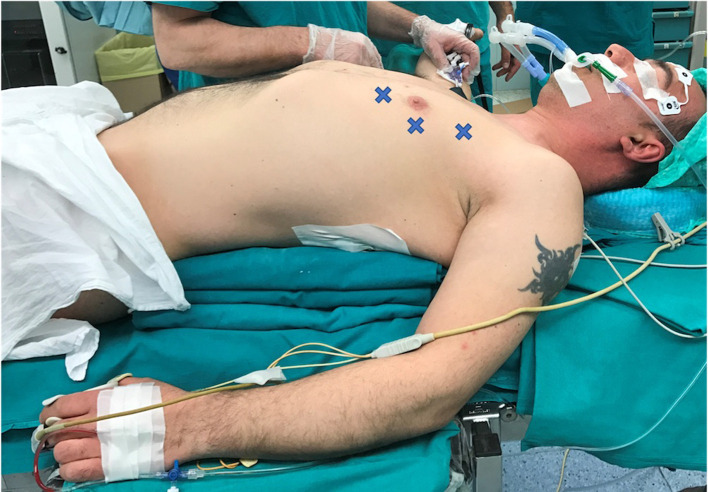
Patient position and trocar placement.

**Figure 2:**
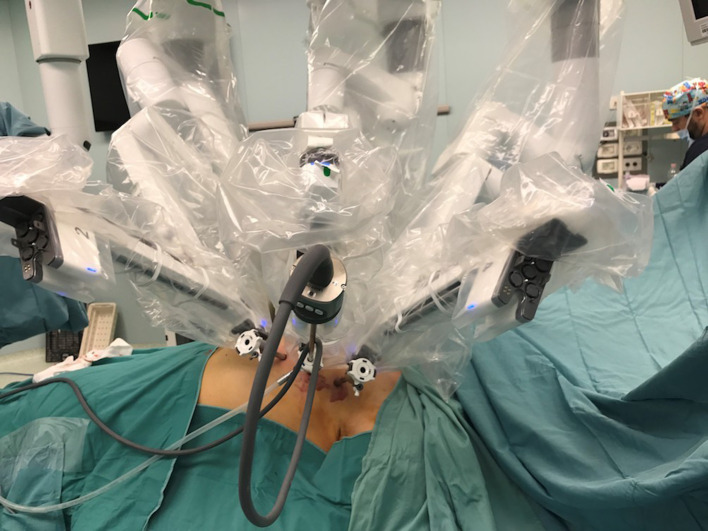
Trocars and robotic arms.

According to the standard robotic technique (12), the patient stood in supine decubitus in a right 30° anti decubitus position, as shown in Figure 1. Since the adoption of the Xi system, three incisions of 8 mm each, one for every trocar placement, were performed. The camera trocar, equipped with an 8-mm camera with a 30° optic, was positioned at the fourth intercostal space on the midaxillary line. The second trocar was placed at the third intercostal space on the anterior axillary line, and the third trocar at the fifth intercostal space between the medioclavicular and the anterior axillary lines (Figure 2). In this setting, no utility trocar for the assistant surgeon is usually placed. The patient's left hemithorax is usually inflated with 7–8 mmHg of CO_2_.

Dissection was performed using a Maryland Bipolar Forceps through the right robotic port and a Fenestrated Bipolar Forceps through the left port.

Thymectomy was performed by removing the thymus gland and the perithymic fat located between the phrenic nerves, the diaphragm, and the cervical thymic horns. The surgical specimen was removed within an Endobag through the lowest incision in the mammary sulcus (enlarged, if necessary, by 1–2 cm).

In this study, for patients’ better postoperative analgesia, an intercostal nerve block was performed, infiltrating ropivacaine in the intercostal spaces corresponding to the incisions.

A 24 or 28 Fr chest tube was inserted in the lowest incision space, with the tip positioned in the anterior mediastinum. The drain was removed when secretions were below 200–250 ml within 24 h, with no signs of pneumothorax at chest x-ray.

### Data Collection

Preoperative clinical variables such as age, gender, American Society of Anesthesiologists (ASA) score, body mass index (BMI), MG, and others were collected and evaluated.

Operative time, docking time, intraoperative complications (such as blood loss and conversion), and postoperative outcomes (i.e., chest tube duration, postoperative hospital stay, and 30-day readmission) were also collected and analyzed.

The docking time was defined as the time interval between the first skin incision and the start of the console time.

The console time was defined as the time that the operating surgeon spent at the console driving robotic arms and performing intrathoracic procedures.

Mortality was defined as death occurring within 30 days of surgery.

### Statistical Analysis

Continuous variables were reported as mean ± standard deviation and compared using the Student’s *t*-test. Categorical variables were analyzed using Fisher's exact or the chi-square test.

Pearson correlation coefficient was used to explore the type of correlation between the ordinal number of consecutive thymectomies and the corresponding operative time of the procedures and then to write the function that better matched the plotting of the surgical times over the series times.

The CUSUM technique of the operative time was used to define the completion of our learning curve (CLC) in RAT.

The CUSUM series was defined as follows: ∑(*X_i_*−*X*_0_), where *X_i_* was an individual measurement [operative time of each case (*n_i_*)] and *X*_0_ was a predetermined reference level, here set as the mean operative time of all cases. The CUSUM series was plotted against the consecutive procedures to calculate the point of downward inflection on the graph or cut-off value [the number of surgical procedures (*n_i_*) to overcome the LC, at which the highest value of ∑(*X_i_*−*X*_0_) was reached].

The cut-off point of the CUSUM score was then used to divide the series into two groups: group A (made up by cases with a progressive number ≤ to the cut-off value) representing the early-experience group (or phase 1 of the LC) and group B (made up by cases with a progressive number > to the cut-off value) representing the late-experience one (or phase 2 of the LC).

The main intraoperative and postoperative outcomes of the two groups were also evaluated.

To eliminate any possible bias related to different surgical times in the case of myasthenic patients, where an accurate removal of all mediastinal tissue is required, myasthenic and non-myasthenic cases were analyzed separately. Moreover, the docking time of the whole series was investigated separately from console time.

Furthermore, a two-sided Bernoulli CUSUM chart was plotted to detect the point of “mastery” of the technique, defined as the point where the operative time became consistent with the mean, without further significant changes in terms of mean operative time (13, 14).

A *p*-value less than 0.05 was considered statistically significant.

Statistical analysis was performed using Microsoft Office Excel and SPSS Statistics for IOS, Version 25.0 (IBM Corp., Armonk, NY, USA).

## Results

A total of 113 consecutive patients were selected for the LC analysis according to our inclusion and exclusion criteria. The main characteristics of the myasthenic and non-myasthenic series are summarized in Table 1.

**Table 1:** Clinicopathological characteristics of patients of the entire cohort.

Variable	Total (*n* = 113)
Preoperative characteristics	
Age (years)	46.05 ± 18.77
Gender (M)	38 (33.6%)
Smoking	56 (49.6%)
ASA score	2.14 ± 0.55
BMI	25.95 ± 3.66
MG	70 (61.9%)
Thymoma in MG	23 (24.4%)
Thymoma	39 (34.5%)
Thymic hyperplasia	72 (63.7%)
Thymic carcinoma	2 (1.8%)
PO_2_ (mmHg)	94.13 ± 13.50
FEV1%	105.72 ± 12.97
Intraoperative results	
Docking time (min)	23.14 ± 6.82
Operative time (min)	186.90 ± 70.23
Side of access (left)	94 (83.2%)
Conversion	3 (2.7%)

*MG, myasthenia gravis*.

Seventy patients (61.9%) were affected by MG and 38 (33.6%) were male. The mean age of the population was 46.05 ± 18.77 years; the mean ASA score and BMI index were 2.14 ± 0.55 and 25.95 ± 3.66, respectively. Seventy-two (63.7%) patients had thymic hyperplasia, while 39 (34.5%) had thymoma and 2 (1.8%) had thymic carcinoma.

Ninety-four (83.2%) patients were operated on by accessing the thorax from the left. The mean console time of the whole series was 186.90 ± 70.23 min (184.30 ± 77.68 min for myasthenic patients vs. 191.14 ± 77.69 for non-myasthenic patients, *p* = 0.617). The mean docking time for the whole cohort was 23.14 ± 6.82 min.

### Results in Myasthenic Patients

**Figure 3:**
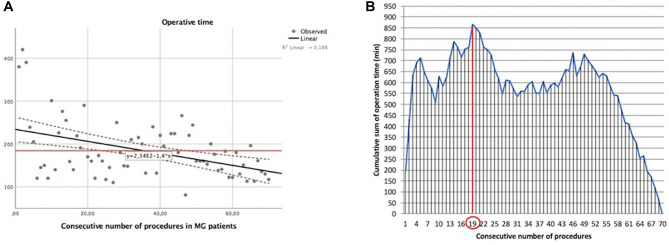
Learning curve of robot-assisted myasthenic thymectomies. (**A**) Correlation between the operation time and the consecutive number of procedures [the red line represents the average time; the Pearson correlation coefficient (*r*) of linear function is −0.433]; (**B**) cumulative sum (CUSUM) plot for the overall surgical time. The red circle is the CLC cut-off value on the plot of CUSUM analysis. CLC, completion of learning curve.

In our experience with MG patients, the duration of surgery and the consecutive number of procedures showed a statistically significant linear correlation (*y* = −1.4*x* + 234). The Pearson correlation coefficient (*r*) was −0.433 with a two-tailed *p* = 0.0002 (Figure 3A).

The CLC cut-off was settled by the point of downward inflection on the plot of CUSUM analysis (Figure 3B) and was observed after 19 patients.

Considering the CLC cut-off of 19 patients, the whole series of myasthenic patients was divided into two groups: phase 1 (first 19 cases of the LC) and phase 2 (the last 51 cases).

The mean operative time in phase 1 was 229.79 ± 93.39 min, while it was 167.35 ± 41.63 min in phase 2, p≪0.001.

Clinical preoperative characteristics of patients belonging to the two phases were similar in terms of age, BMI, ASA score, and other parameters, as shown in Table 2.

**Table 2:** The clinicopathological characteristics and postoperative outcomes of MG patients stratified by the completion of the learning curve (CLC) cut-off.

Variable	CLC cut-off		*p*
	Phase 1 (*n* = 19)	Phase 2 (*n* = 51)	
Preoperative characteristics			
Age (years)	42.21 ± 17.53	40.98 ± 18.08	0.799
Gender (M)	3 (15.78%)	18 (35.29%)	0.113
Smoking	10 (52.53%)	23 (45.09%)	0.574
ASA score	2.00 ± 0.00	2.42 ± 0.50	**0.003**
BMI	25.38 ± 3.95	27.00 ± 2.83	0.619
Thymic hyperplasia	14 (73.68%)	31(60.78%)	0.534
Thymoma	4 (21.05%)	20 (39.21%)	
Thymic carcinoma	1 (5.26%)	0 (0%)	
Intraoperative results			
Operative time (min)	229.79 ± 93.39	167.35 ± 41.63	≪0.001
Side of access (left)	17 (89.47%)	46 (90.19%)	0.929
Intraoperative complications	0 (0%)	0 (0%)	1.00
Blood loss (ml)	28.95 ± 12.97	33.43 ± 45.06	0.672
Conversion	0 (0%)	1 (1.9%)	0.539
Postoperative outcomes			
Chest tube duration	2.68 ± 0.67	2.48 ± 0.92	0.382
Postoperative hospital stay	3.42 ± 1.07	3.78 ± 2.35	0.520
Thirty-day readmission	2 (10.53%)	0 (0%)	**0.020**
Thirty-day mortality	0 (0%)	0 (0%)	1.00

*Bold indicates p-value inferior to 0.01.*

There was only one conversion to open surgery in phase 2 (vs. 0 conversions in phase 1, *p* = 0.539) because of vascular infiltration. The incidence of intraoperative complications was null in both groups. There were also no differences between the two phases of the LC in terms of blood loss (*p* = 0.672), duration of postoperative drainage (*p* = 0.382), and length of postoperative stay (*p* = 0.520), as shown in Table 2. However, a significantly higher hospital readmission at 30 days for pleural effusion was recorded in two myasthenic patients operated on during the first phase of the LC (2 cases vs. 0, *p* = 0.02).

The main results of both phases of the LC for MG patients stratified by CLC cut-off are shown in Table 2.

### Results in Non-Myasthenic Patients

**Figure 4:**
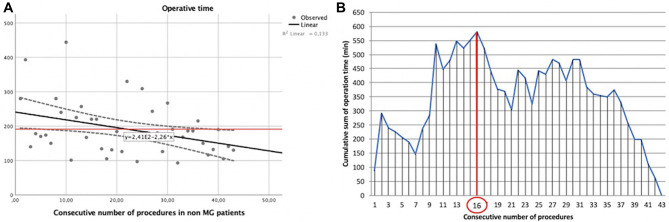
Learning curve of robot-assisted non-myasthenic thymectomies. (**A**) Correlation between the operation time and the consecutive number of procedures [the red line represents the average time; the Pearson correlation coefficient (*r*) of linear function is −0.365]; (**B**) cumulative sum (CUSUM) plot for the overall surgical time. The red circle is the CLC cut-off value on the plot of CUSUM analysis. CLC, completion of learning curve.

The same analysis was performed for non-myasthenic patients who had undergone robotic surgery for thymic lesions (Table 1). In this case, operative times and the consecutive number of procedures were correlated by the following linear function: *y* = −2.26*x* + 241, with a Pearson correlation coefficient (*r*) of −0.365 and a two-tailed *p* = 0.016 (Figure 4A).

The CLC cut-off on the plot of CUSUM analysis (Figure 4B) was 16 cases.

Again, non-myasthenic patients were divided into two groups: phase 1 (the first 16 cases of the LC) and phase 2 (the last 27 cases).

The mean operative time in phase 1 was 277.44 ± 90.50 min, while it was 169.63 ± 61.10 min in phase 2, *p* = 0.016.

The main preoperative characteristics of non-myasthenic patients in the two phases were superimposable (Table 3). The conversion was related to the dimension of thymoma in phase 1 and vascular infiltration in phase 2. There was no intraoperative complication in both groups. There was no difference in terms of 30-day readmission rate (1 patient with pneumothorax vs. 2 patients with pleural effusion, *p* = 0.885) blood loss, and postoperative stay, as shown in Table 3.

**Table 3:** The clinicopathological characteristics and postoperative outcomes of non-MG patients stratified by the completion of the learning curve (CLC) cut-off.

Variable	CLC cut-off		*p*
	Phase 1 (*n* = 16)	Phase 2 (*n* = 27)	
Preoperative characteristics			
Age (years)	56.44 ± 15.17	52.19 ± 19.42	0.458
Gender (M)	8 (50.0%)	9 (33.33%)	0.280
Smoking	8 (50.0%)	15 (55.55%)	0.724
ASA score	1.83 ± 0.72	2.11 ± 0.58	0.254
BMI	27.02 ± 5.71	25.00 ± 0.00	0.668
Thymic hyperplasia	9 (56.25%)	18 (66.66%)	0.483
Thymoma	6 (37.5%)	9 (33.33%)	
Thymic carcinoma	1 (6.25%)	0 (0%)	
Intraoperative results			
Operative time (min)	227.44 ± 90.54	169.63 ± 61.09	**0.016**
Side of access (left)	13 (81.25%)	18 (66.66%)	0.303
Intraoperative complications	0 (0%)	0 (0%)	1.00
Blood loss (ml)	25.63 ± 8.14	29.07 ± 16.53	0.441
Conversion	1 (6.25%)	1 (3.7%)	0.702
Postoperative outcomes			
Chest tube duration	2.94 ± 1.34	2.67 ± 3.13	0.745
Postoperative hospital stay	3.63 ± 2.16	4.26 ± 4.69	0.614
Thirty-day readmission	1 (6.25%)	2 (7.4%)	0.885
Thirty-day mortality	0 (0%)	0 (0%)	1.00

*Bold indicates p-value inferior to 0.01.*

**Figure 5:**
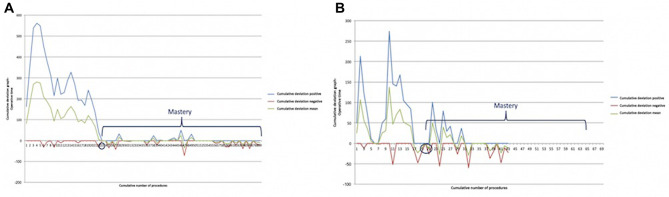
Bernoulli cumulative deviation curves for CUSUM. (**A**) Myasthenic patients; (**B**) non-myasthenic patients. The blue circle is the point where mastery was reached.

Mastery was reached after 23 procedures in myasthenic patients (Figure 5A) and after 20 in non-myasthenic patients (Figure 5B).

The intraoperative and 30-day mortality were null in both phases of the LC of myasthenic and non-myasthenic groups.

### Results for Docking Time

Lastly, a further analysis was performed on docking time for the whole series of 113 cases.

**Figure 6:**
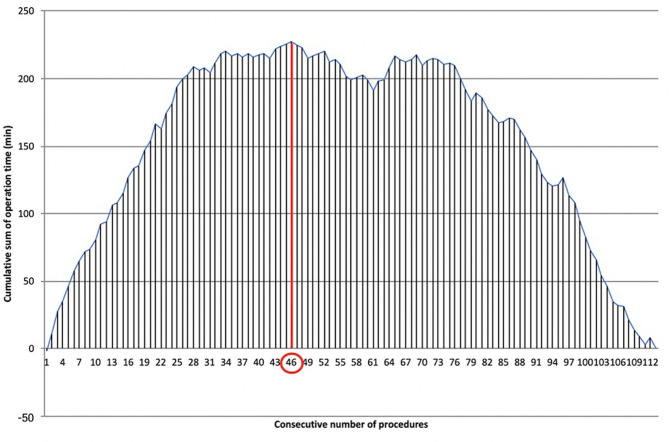
Learning curve of robotic docking time. Cumulative sum (CUSUM) plot for the overall docking time. The red circle is the CLC cut-off value on the plot of CUSUM analysis. CLC, completion of learning curve.

The LC was overcome for docking time after 46 cases (Figure 6), recording a significant reduction of time after the first phase (28.09 ± 5.37 min vs*.* 19.75 ± 5.51 min, p≪0.001). Mastery in docking was reached after 67 procedures.

Docking times longer than the mean times seemed to be not related to patients’ BMI (*p* = 0.220) or the less-usual approach of the thorax from the right (*p* = 0.705).

## Discussion

LC evaluation is one of the main areas of surgical research in the field of minimally invasive procedures in thoracic surgery, especially in robotics, which represents the last frontier of minimally invasive thoracic surgery approaches.

The identification of the learning phase of this technique is crucial, as it allows recommendations for when and how to establish a training program to be made to avoid potential harm to patients and allow the supervision of surgeons not experienced in the technique.

To date, only three studies focusing on the LC of RAT have been produced, evaluating relatively small groups of patients ranging from 9 to 70 patients. Huang et al. (8) evaluated a group of 23 patients and reported a mean operative time of 105.3 min in the first 10 cases and 80.4 min in the last 10 cases (*p* < 0.05).

A smaller observational study by Ro et al. also illustrated a drastic LC in the initial robotic cases (9). The first five performed cases had a mean operative time of 282 min, with the next four cases having a mean operative time of 118 min (*p* = 0.014).

Kamel et al. reported the largest series of 70 patients undergoing thymectomy for MG (10). The included RAT cases were performed by four different surgeons. Operative time and estimated blood loss were used as surrogates of technical proficiency. A plateau point was observed following surgeons’ initial 15–20 cases. Furthermore, robotic cases were stratified into two groups according to the number of cases performed by each surgeon (early 15 cases vs. late 15 cases). The late group had a shorter operative time (94 vs. 107 min, *p* = 0.018), with increasing use of the left-side approach (64% vs. 27%, *p* = 0.002). There were no differences in estimated blood loss, intraoperative complications, conversion rate, postoperative complications, or length of stay between the two groups.

Overall, previous studies reported an ascending LC with regard to operative time, consisting of the first 10–20 cases.

Our study, presenting a detailed single-surgeon LC analysis for RAT, differs from the previous reports in several ways. First, it did not assess the CLC analyzing raw timing data, but it applied the CUSUM analysis, that is, a well-established method to represent data from consecutive procedures, transforming the variability of raw data into a CUSUM of differences between each value and their mean. Then, the intra and early postoperative results before and after CLC were compared.

Moreover, our analysis is based on a “pure learning curve” based on a consecutive series of 113 patients operated on by a single thoracic surgeon (E.M.), in order to reduce any bias related to different previous experiences or personal skills belonging to different surgeons. Indeed, even in the previous paper with the widest experience (10), the operative time analyzed referred to four different surgeons. In the other two papers (8, 9) the authors did not specify the number of operative surgeons involved in the study, but the number of patients enrolled ranged from 9 to 23. Therefore, to the best of our knowledge, our single-surgeon experience evaluating the LC in RAT is the widest in the literature.

Furthermore, we decided to evaluate the CLC of RAT in myasthenic and non-myasthenic patients separately. The rationale of this choice is the different approach to the resection of perithymic fat tissue. Although our surgical treatment for non-myasthenic thymomatous patients is represented by a radical thymectomy with a resection of the mediastinal fat tissue, we do not “extremize” the dissection by skeletonizing the contralateral phrenic nerve to avoid unnecessary maneuvers that may affect the integrity of the abovementioned nerve. On the other hand, radical thymectomy for thymomatous or non-thymomatous myasthenic patients requires a “maniacal” dissection of all mediastinal fat tissue between the phrenic nerves, neck, and diaphragm (15), making the surgical procedure technically more challenging and inevitably longer. Our results confirmed a longer CLC in myasthenic patients (19 cases) compared with non-myasthenic patients, where CLC was achieved after 16 cases. Similar behavior was shown by the point of mastery, where the operative time became consistent with the mean, without further significant changes in mean operative time: it was reached after 23 procedures in myasthenic patients and earlier, after 20 procedures, in the non-myasthenic population.

The CLC of docking time was analyzed separately from the operative CLC. Port placement and angulation, adequate insufflation, robot positioning, and docking are all key factors that make the difference between a straightforward operation and one complicated by poor visibility or clashing instruments. We decided to separate the robotic docking time from the “pure” surgical time because the thoracic robotic program at Fondazione Policlinico Universitario A. Gemelli IRCCS started immediately after the introduction of the robotic platform, and the whole operating room (OR) staff started the robotic training contemporarily. Therefore, it was feasible to analyze the two different LCs separately. Docking CLC was achieved after 46 cases, while docking mastery was achieved after 67 cases, therefore requiring many more procedures to reach mastery than the “pure” surgical CLC. This may reflect the need to “wait” for the CLC of multiple nurses shifting in the OR.

Analyzing our intra and postoperative results, we found a statistically significant reduction in operative time after CLC in myasthenic patients (*p* < 0.001), non-myasthenic patients (*p* = 0.01), and robotic docking (*p* < 0.001). The statistically significant operative time reduction (console and docking time) after CLC validates the efficacy of CUSUM analysis in our experience.

Contrary to what was reported in other studies (16) where an analysis of complications before and after CLC calculated by CUSUM analysis was performed, the very low or null incidence of intra and postoperative complications in our experience prevented us from evaluating the variation of surgical outcomes along our LC. On the other hand, the similar outcomes obtained before and after the CLC in our series show that RAT is a safe procedure, as previously reported (17), even at the beginning of the LC.

The results obtained in our study, based on a validated mathematical model, evaluating the LC of a single surgeon who operated on a homogeneous cohort of patients, confirm the CLC between 15 and 20 operations as suggested by previous experiences (8–10).

## Conclusions

The CLC for the surgical time was obtained after the 16th procedure in non-myasthenic patients and after the 19th in myasthenic patients. The robotic docking CLC was obtained after 46 cases.

According to our data, the LC in RAT seems to be steep, and RAT is confirmed as safe even before CLC.

Although this study included a reasonable number of procedures, a greater number of cases would be even more useful to identify oscillations in the surgical performance due to other causes not evaluable in this cohort.

## Data Availability

The original contributions presented in the study are included in the article/supplementary material, further inquiries can be directed to the corresponding author/s.
